# Transcriptional Coactivators: Driving Force of Plant Immunity

**DOI:** 10.3389/fpls.2022.823937

**Published:** 2022-01-28

**Authors:** Muhammad Saad Shoaib Khan, Faisal Islam, Huan Chen, Ming Chang, Daowen Wang, Fengquan Liu, Zheng Qing Fu, Jian Chen

**Affiliations:** ^1^International Genome Center, Jiangsu University, Zhenjiang, China; ^2^Institute of Crop Science and Zhejiang Key Laboratory of Crop Germplasm, Zhejiang University, Hangzhou, China; ^3^Institute of Plant Protection, Jiangsu Academy of Agricultural Sciences, Nanjing, China; ^4^Department of Biological Sciences, University of South Carolina, Columbia, SC, United States; ^5^The Key Laboratory of Bio-interactions and Plant Health, College of Life Science, Nanjing Agricultural University, Nanjing, China; ^6^State Key Laboratory of Wheat and Maize Crop Science and College of Agronomy, Henan Agricultural University, Zhengzhou, China

**Keywords:** salicylic acid, NPR1, EDS1, mediator, CDK8, transcriptional coactivators, plant immunity

## Abstract

Salicylic acid (SA) is a plant defense signal that mediates local and systemic immune responses against pathogen invasion. However, the underlying mechanism of SA-mediated defense is very complex due to the involvement of various positive and negative regulators to fine-tune its signaling in diverse pathosystems. Upon pathogen infections, elevated level of SA promotes massive transcriptional reprogramming in which Non-expresser of PR genes 1 (NPR1) acts as a central hub and transcriptional coactivator in defense responses. Recent findings show that Enhanced Disease Susceptibility 1 (EDS1) also functions as a transcriptional coactivator and stimulates the expression of *PR1* in the presence of NPR1 and SA. Furthermore, EDS1 stabilizes NPR1 protein level, while NPR1 sustains *EDS1* expression during pathogenic infection. The interaction of NPR1 and EDS1 coactivators initiates transcriptional reprogramming by recruiting cyclin-dependent kinase 8 in the Mediator complex to control immune responses. In this review, we highlight the recent breakthroughs that considerably advance our understanding on how transcriptional coactivators interact with their functional partners to trigger distinct pathways to facilitate immune responses, and how SA accumulation induces dynamic changes in NPR1 structure for transcriptional reprogramming. In addition, the functions of different Mediator subunits in SA-mediated plant immunity are also discussed in light of recent discoveries. Taken together, the available evidence suggests that transcriptional coactivators are essential and potent regulators of plant defense pathways and play crucial roles in coordinating plant immune responses during plant–pathogen interactions.

## Introduction

Plants are constantly exposed to a wide range of destructive pathogens that cause dreadful diseases and considerably reduce crop yield by 10–40% (Yadav and Srivastava, 2017; Savary et al., 2019). To cope with these challenges, plants have developed a multilayered immune system that is highly efficient in the prevention of pathogen infections. Plant defense is an extremely complex and tightly regulated process that involves regulations at the transcriptional level (Yuan et al., 2021). These signaling cascades are activated after the recognition of pathogenic microbes. The pathogen-associated molecular patterns (PAMPs) are recognized by plasma membrane-localized leucine-rich repeat (LRR) receptor kinases or receptor-like proteins to trigger a multifaceted basal immune response, known as PAMP-triggered immunity (PTI; Chen et al., 2021b). To enhance pathogenicity for successful establishment of growth, plant pathogens secrete effectors to compromise PTI. To combat this, plants have evolved sophisticated mechanisms for the recognition of pathogen effectors or their actions on host targets and induce a more effective and robust resistance response known as effector-triggered immunity (ETI; Martel et al., 2021).

Effector-triggered immunity activates strong defense responses that lead to programmed cell death (PCD; including swelling of mitochondria, ROS generation, enlargement of central cell vacuole, rupturing of the plasma membrane, and shrinkage of protoplast), which completely inhibits pathogen colonization at the infection site and is known as the hypersensitive response (HR; Betsuyaku et al., 2018; Peng et al., 2018; Liu et al., 2020a). ETI is mainly regulated by intracellular immune receptors known as nucleotide-binding (NB) LRR receptors (NLRs). According to the presence of coiled-coil (CC), Toll/interleukin-1 receptor (TIR), or Resistance to Powdery Mildew 8 (RPW8) domains at the N-terminus, plant NLRs are divided into three subgroups: CNLs (CC-NLRs), TNLs (TIR-NLRs), and RNLs (RPW8-NLRs; Chen et al., 2021c). CNLs and RNLs are considered as “sensor NLRs” and could directly or indirectly detect the presence of pathogen effectors and activate immune responses (Jones et al., 2016). Several lines of evidence suggest that NLRs are responsible for the recognition of pathogen effectors, and this recognition is the first step of immunity activation, whereas the actual process of stimulation of ETI needs other signaling components (Saleem et al., 2021). The coordinated action of ETI stimulates mitogen-activated protein kinase signaling, oxidative stress, the expression of *pathogenesis-related* (*PR*) genes, and the production of salicylic acid (SA). High level of SA will then induce the generation of mobile signals to trigger systemic acquired resistance (SAR) at the distal parts of the plants (Fu and Dong, 2013; Saleem et al., 2021).

Under biotrophic and hemibiotrophic pathogens attacks, SA accumulation and signaling cascade are primarily regulated by Enhanced Disease Susceptibility 1 (EDS1) and Non-expresser of PR genes 1 (NPR1), which act as transcriptional coactivators, to activate defense-related pathways to establish plant immunity (Li et al., 2019a). Transcriptional coactivator works together with other partners to positively regulate the transcription of certain genes (Jin et al., 2018). Multiple transcriptional coactivators are essential for transcriptomic reprogramming in SA-dependent plant immunity. Despite the growing body of evidence demonstrating their dynamic participation in defense responses, underlying processes related to their activation, regulation, pre/post-transcriptional and translational modifications, and interactions are still largely unknown (Jin et al., 2018; Yu et al., 2021). Over the past few decades, considerable advancement has been made in elucidating SA-mediated immune signaling at both molecular and cellular levels. Here, we summarize recent literature revealing the details of an emerging role of transcription coactivators, such as NPR1, EDS1, and Mediators, in the context of plant immunity. In addition, we also discuss recent breakthroughs in the field that could provide a mechanistic understanding of functional interactions between plant immunity and regulators of SA signaling at different levels.

## Salicylic Acid Biosynthesis and its Functions in Plant Immunity

**Figure 1:**
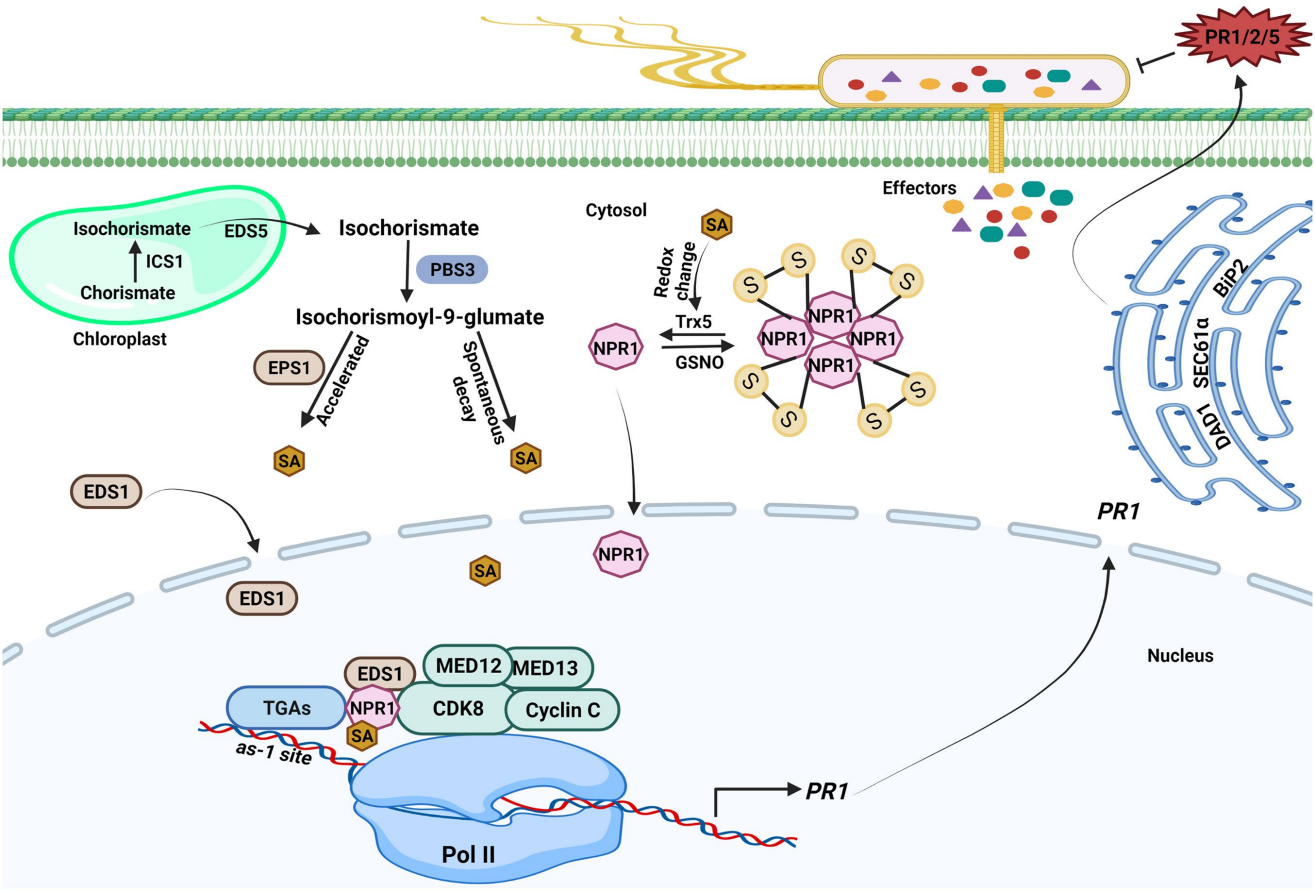
Transcriptional regulations of salicylic acid-mediated plant immunity. Upon pathogen infection, salicylic acid (SA) generation is initiated in the chloroplast. ICS1 catalyzes the conversion of chorismate to isochorismate, and then, isochorismate is exported to the cytosol by Enhanced Disease Susceptibility (EDS5) 5. PBS3 catalyzes the conjugation of L-glutamate to isochorismate in the cytosol, resulting in isochorismate-9-glutamate. SA is produced from isochorismate-9-glutamate through spontaneous decay. The EPS1 acts as an isochorismate-9-glutamate pyruvoyl-glutamate lyase that could also break down N-pyruvoyl-L-glutamate to create SA. The *NPR1* gene expression is aided by SA because it encourages the interaction between the WRKY transcription factors and NPR1, which brings cyclin-dependent kinase 8 (CDK8) to the *NPR1* promoter’s W-box and enables gene expression. The CDK8 kinase module mediators are also implicated in *PR1* gene expression. SA also promotes redox changes, which reduces NPR1 oligomers to monomers. The monomeric NPR1 molecule travels from the cytosol to the nucleus and activates downstream NPR1-dependent genes. NPR1 physically interacts with other coactivators, such as Enhanced Disease Susceptibility (EDS1) 1 and CDK8, to positively regulate the expression of *PR* genes expression in an SA-dependent environment. The *PR1* mRNA is further exported from the nucleus to the cytosol to produce PR1 protein. ER-resident genes (*SEC61α*, *DAD1*, and *BiP2*) govern the secretion of PR proteins into the apoplast to counter pathogen. The figure was created with BioRender.com.

The plant hormone SA is a phenolic compound that plays a critical role in regulating immune responses. Studies have shown that pathogen infection increases SA level; SA is essential for SAR establishment and acts as a vital modulator of plant immunity (Chen et al., 2020). In *Arabidopsis thaliana*, approximately 90% of pathogen-induced SA is synthesized by the isochorismate pathway; two *isochorismate synthase* (*ICS*) genes *ICS1* and *ICS2* are found in the *Arabidopsis* genome, although only *ICS1* is rapidly induced by pathogens (Wildermuth et al., 2001). Pathogen-induced SA accumulation and SAR were abolished when *ICS1* was knocked out. ICS1 converts chorismate to isochorismate in the plastid, and Enhanced Disease Susceptibility 5 (EDS5) transports isochorismate into the cytoplasm, where it is further metabolized to produce SA *via* the action of PBS3 and EPS1 (Figure 1; Wildermuth et al., 2001; Torrens-Spence et al., 2019). PBS3, as a GH3 acyl adenylase-family enzyme, catalyzes the conjugation of L-glutamate to isochorismate in the cytosol to generate isochorismate-9-glutamate, which is then used to produce SA through spontaneous decay. EPS1 functions as a BAHD acyltransferase-family protein, which could break down N-pyruvoyl-L-glutamate to generate SA.

Apart from playing a critical role in the regulation of SAR signaling, SA also amplifies PTI signaling. It was revealed in a recent study that after pathogen attack, the transcription of the early PAMP marker genes was significantly reduced in the SA receptor mutant *npr1-2* (Chen et al., 2017). SA contributes to activation of the genes that function both upstream and downstream of PAMP receptors (Ding et al., 2018). SA serves dual functions in ETI. According to an early finding, the *A. thaliana* SA-deficient *NahG* transgenic lines are more vulnerable to the avirulent bacterial pathogen *Pseudomonas syringae* pv*. tomato* carrying *avrRpt2* (Delaney et al., 1994), suggesting that initiation of ETI requires SA signaling. Consistently, when ETI is activated in *Arabidopsis* by the *Pseudomonas* effector AvrRpm1 or AvrRpt2, local SA content is remarkably elevated, with the elevated SA concentration associated with HR or PCD (Nawrath and Metraux, 1999). Additionally, artificial enhancement of SA signaling has been shown to negatively affect cell death during ETI. Devadas and Raina (2002) demonstrated that the *P. syringae* pv. *maculicola* ES4326 strain harboring *avrRpm1* failed to elicit HR in the *Arabidopsis* Col-0 plants pre-treated with SA. Rate and Greenberg (2001) reported that the NPR1-overexpressing *Arabidopsis* plants exhibited a reduced HR response, while the *npr1* mutants displayed a more severe HR, in the infection assays conducted with *P. syringae* carrying the *avrRpm1* gene. These studies suggest that SA is a multifaceted phytohormone involved in various signal transduction systems in plant immune responses (Li et al., 2019a).

## Roles of Mediator in the Transcriptional Regulations of Plant Immunity

### The Functional and Modular Organization of Mediator

Plants have evolved a substantial number of transcription factors (TFs) to coordinate and to fine-tune complex transcriptional programs (Malik et al., 2020). For example, the *Arabidopsis* genome has about 1,500 transcription factors, which may form diverse protein complexes to orchestrate different gene expression patterns in various signaling cascades (Sinha and Kumar, 2021). As a multi-protein complex, Mediator connects DNA-binding TFs with RNA polymerase II (Pol II) and serve as a central hub to regulate diverse aspects of transcription (Malik and Roeder, 2010). The initiation, elongation, and termination steps of mRNA synthesis are catalyzed by RNA Pol II, which is modulated by specific transcription factors and the Mediator complex.

**Figure 2:**
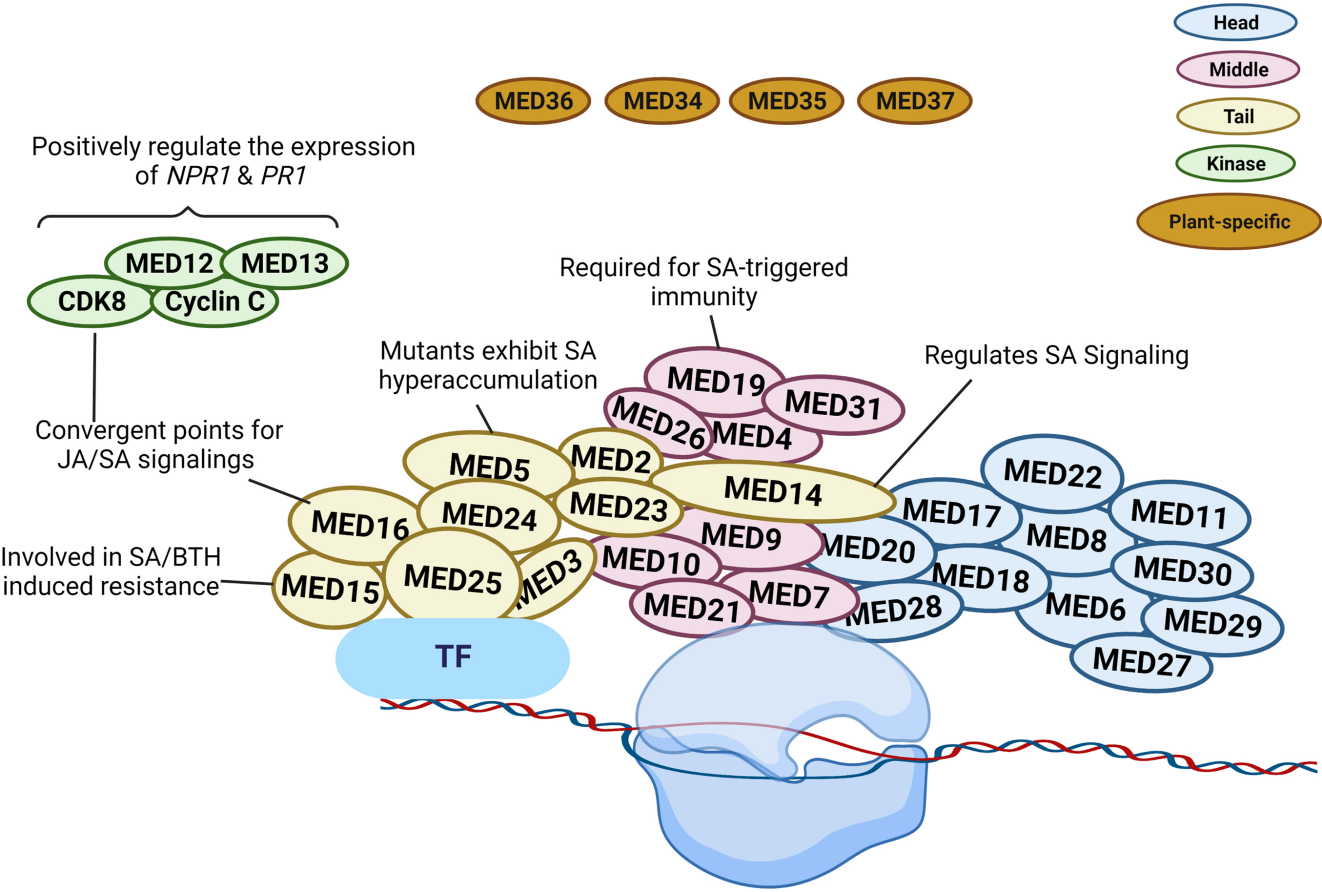
Schematic illustration of plant Mediator structure and function. The Mediator complex in *Arabidopsis* is categorized into the following modules: Head (blue), Middle (pink), Tail (yellow), kinase (green), and plant-specific subunit (brown). The localization of the individual subunit within each module is arbitrary. The head and middle Mediator modules are attached to RNA polymerase II (Pol II), whereas the tail submodule interacts with certain transcription factors (TFs). Mediators connect DNA-binding TFs with the Pol II and serve as a bridge to regulate diverse aspects of transcription. Some Mediators with known functions in SA signaling are represented in the diagram. The figure was created with BioRender.com.

According to structural studies, the overall structure of the Mediator complex may be categorized into three major modules (Head, Middle, and Tail). The head and middle modules interact with Pol II, while the tail module interacts with various transcription factors (Larivière et al., 2012; Verger et al., 2019; Malik et al., 2020). According to previous studies, *Arabidopsis* consists of four (MED34, MED35, MED36, and MED37) plant-specific subunits of Mediator (Figure 2). However, Guo et al. (2021) carried out the affinity purification and mass spectrometry analysis of these four Mediator subunits and found that these could not be co-purified with other Mediator subunits, so they believe that MED34, MED35, MED36, and MED37 should not be regarded as Mediator subunits of *Arabidopsis*.

### Mediator Middle Module

In transcriptional regulation, the various Mediator subunits can function as coactivator or co-repressor. Gene expression can be altered due to the involvement of certain Mediator subunits in the epigenetic and architectural modifications of chromatin (Lai et al., 2013). MED19 is a Mediator subunit located in the middle module. It assists the defense mechanism of plants *via* regulating the gene expression in SA, jasmonic acid (JA), and ethylene (ET) signaling pathways (Figure 2). Caillaud et al. (2013) reported the interaction between the HaRxL44 effector protein from the oomycete pathogen *Hyaloperonospora arabidopsidis* and host Mediator component MED19a. This effector destabilizes MED19 at the protein level and consequently results in the downregulation of SA marker genes (*PR1*, *PR2*, *PR5*, and *WRKY70*). Furthermore, *med19a* mutants show reduced SA-triggered immune responses, whereas overexpression of MED19a promotes SA-mediated plant defense. The presence of the HaRxL44 effector or the lack of MED19a in plants was linked to lower *PR1* expression. SA-induced *PR1* expression was similarly shown to be decreased in *med19a* mutants but increased in *MED19a* overexpressing lines. In *HaRxL44* overexpression lines and *med19a* mutants, JA/ET marker genes, such as *PDF1.2*, *JAZ1*, and *JAR1*, were activated. As a result of HaRxL44-mediated MED19a degradation, SA-dependent transcription was altered, and the balance between the JA/ET and SA pathways was disrupted. These findings imply that MED19a is a positive regulator of SA-triggered immunity against biotrophic pathogens and is engaged in SA/JA crosstalk (Caillaud et al., 2013).

### Mediator Tail Module

MED14 is a component of the Mediator tail module and has been found to regulate various plant development and pathogen defense processes. In rice, the knockdown of *OsMED14* showed pleiotropic effects (fewer panicles, reduced plant height, lower pollen fertility, and narrow leaves, etc.; Malik et al., 2020). In addition, MED14 controls immune responses to pathogen infection *via* regulating SA signaling in plants. Zhang et al. (2013) evaluated the T-DNA insertion mutant of *MED14* and found that *med14* mutants were susceptible to the avirulent pathogen *Pst* DC3000 carrying *avrRpt2*. The mutation of *med14* resulted in the inhibition of the NAD^+^-mediated *PR1* gene expression. In complementation lines, NAD^+^ driven *PR1* gene expression was restored, supporting the significance of MED14 in extracellular NAD^+^-mediated signaling (Zhang et al., 2013). After pathogen infection, the *med14-1* mutants showed reduced resistance to *Pst* DC3000 carrying *avrRpt2*, and several genes were differentially regulated between wild-type and *med14* mutant plants. Importantly, the master regulator of SA signaling, *NPR1*, was significantly downregulated in *med14* mutants. It is also worth noting that activation of the SA biosynthesis genes *ICS1*, *EDS5*, and *PBS3* requires functional MED14 (Zhang et al., 2013). Furthermore, compared to *med16* mutants, the transcriptional alterations in response to *Pst* DC3000 carrying *avrRpt2* in *med14-1* mutant plants demonstrated differential expression in numerous genes associated with SAR and NPR1, showing that MED14 and MED16 use distinct mechanisms to regulate the SA signaling and SAR pathways (Figure 2; Zhang et al., 2013).

Another Mediator tail module subunit in *Arabidopsis*, MED15, was first discovered as NRB4 (non-recognition-of-BTH4) during mutant screening (Canet et al., 2012). The *med15* mutant showed unresponsiveness toward an analog of SA, benzothiadiazole (BTH), similarly to the *npr1-1* mutant exhibiting insensitivity to SA. When exposed to SA or BTH, the wild-type plants but not the *med15* or *npr1* mutants developed increased resistance to *Pst* DC3000. In addition to that, the *Pst* DC3000 or BTH treatment did not affect the PR1 protein level in *med15* or *npr1* mutants. However, yeast two-hybrid analysis showed no interaction between NPR1 and MED15, even in the presence of SA. Moreover, the overexpression lines of *MED15* displayed an enhanced response to SA (Figure 2). Canet et al. (2012) showed that MED15 may act downstream of NPR1 to control SA-mediated responses.

MED16 is also a subunit in the tail module of Mediator complex and was first identified during a screen for mutants vulnerable to acclimatization under cold stress conditions. Thus, this gene was named SENSITIVE TO FREEZING 6 (Warren et al., 1996). Wathugala et al. (2012) reported that mutation of *MED16* led to susceptibility to *P. syringae* and lower *PR1*, *PR2*, and *PR5* mRNA levels compared to Col-0, indicating that MED16 is required for the regulation of SA-induced *PR* genes expression. Another research group suggested a crucial role of MED16 in basal resistance against the necrotrophic fungus *Sclerotinia sclerotiorum*, as the *med16* mutant displayed compromised resistance response (Wang et al., 2015). Further analysis uncovered that the *med16* plants not only showed reduced *NPR1* expression but also exhibited lower expression of JA/ET-responsive genes (Figure 2).

A further tail module component of Mediator is MED5/MED33. The *med5a*/*med5b* mutant hyper-accumulates phenylpropanoids, indicating that MED5 plays a key role in phenylpropanoid homeostasis (Bonawitz et al., 2012). In contrast, a single amino acid alteration in MED5b (i.e., mutant ref4-3) could turn MED5b into a repressor of the pathway, leading to a lower accumulation of phenylpropanoids, dwarfism, and reduced lignin contents (Bonawitz et al., 2012). In *ref4-3*, the expression of defense genes, such as *ICS1*, *PR1*, *PR2*, and *PR5*, was elevated. Furthermore, there was an increased accumulation of free SA and SA conjugates in *ref4-3* (Mao et al., 2019). However, the upregulation of SA signaling genes in *ref4-3* was changed in the *ref4-3*/*cdk8-1* double mutant, resulting in suppressed expression of SA-responsive defense genes and reduced SA accumulation. Consequently, the cyclin-dependent kinase 8 (CDK8) kinase activity was required for SA hyperaccumulation in the *ref4-3* mutant (Figure 2). CDK8 does not physically interact with MED5 according to the structural analysis of the yeast Mediator complex. Hence, future research is required for achieving a better understanding of the functional association between the MED5 and CDK8 subunits in regulating SA signaling.

### CDK8 Kinase Module

The CDK8 kinase module participates actively in transcriptomic reprogramming during plant response to pathogen infection (Mao et al., 2019; Liu et al., 2020b). CDK8 has positive transcriptional regulatory activities in SA-mediated immunity *via* phosphorylating RNA Pol II at the CTD site (Wang and Chen, 2004; Chen et al., 2019). Zhu et al. (2014) discovered that CDK8 promotes *Arabidopsis* resistance to *Alternaria brassicicola* by directly regulating the transcription of *AGMATINE COUMAROYLTRANSFERASE* (*AACT1*). AACT1 participates in the production of a family of secondary metabolites known as hydroxycinnamic acid amides, which have been implicated in fungal resistance (Zhu et al., 2014). As a result, *Arabidopsis* plants are unable to elicit essential defensive responses in the absence of CDK8. MED12 and MED13, two subunits of the CDK8 module, share the same structural and functional roles as CDK8. In *Arabidopsis*, MED12 and MED13 are involved in positive gene regulation under specific circumstances and promote the initial stages of gene transcription (Liu et al., 2020b). Huang et al. (2019) demonstrated that *cdk8* mutants grown under normal conditions showed reduced expression of *ICS1* and *EDS5*, indicating that CDK8 is involved in the transcriptional control of these SA production and transport genes (Figure 2). Similarly, *med12* mutants exhibited lowered SA levels and hampered SAR. The mutation in *MED12* resulted in similar defects, such as lower SA level and weakened SAR (Huang et al., 2019).

The subsequent study by Chen et al. (2019) demonstrated that NPR1 interacts with CDK8 and WRKY transcription factors, such as WRKY18, to promote the expression of *PR* genes in *Arabidopsis*, thereby promoting defense responses. The *cdk8* mutant had lower levels of *NPR1* and *NPR1*-dependent defense gene expression as compared to WT control. CDK8 regulates *NPR1* expression by interacting with WRKY6 and WRKY18 at *NPR1*’s promoter. TGA5 and TGA7 are transcription factors associated with the *PR1* promoter and work together with CDK8 to regulate *PR1* gene expression. This study also discovered that CDK8 recruits RNA Pol II to *NPR1* and *PR1* promoters and coding regions to increase the expression of those genes (Chen et al., 2019). Thus, the contribution of CDK8 to SA-mediated plant immunity was further established as CDK8 promotes functional interactions among NPR1, TGA, TFs, and RNA Pol II under the influence of SA to facilitate *PR1* gene expression.

## HAC1 and HAC5: Loosening Up Chromatin

Histones are alkaline proteins in the nuclei of eukaryotic cells that function to package DNA into structural components known as nucleosomes (Yang et al., 2020). The addition or removal of acetyl and methyl groups to the histone tails, which protrude from the nucleosome core, might alter the physical accessibility of DNA to cell’s transcriptional machinery. Histone acetylation reduces positive charges from histone proteins by adding an acetyl group to the lysine residues, lowering histone-DNA binding, leading to chromatin de-condensation and gene activation (Kumar et al., 2021). The combined actions of several histone acetyltransferases (HATs or HACs) and histone deacetylases constantly regulate the level of histone acetylation (Yang et al., 2020). HATs are divided into four groups based on sequence similarity and domain organization; GNAT (General Control Non-depressible 5-related Acetyltransferase), MYST (MOZ-YBF2/SAS3-SAS2/TIP60), CBP (cAMP-Responsive Element Binding Protein), and TAFII250 (TATA-binding protein Associated Factor; Hu et al., 2019). The abbreviations HAG, HAM, HAC, and HAF are used to represent these families (Pandey et al., 2002).

Histone acetyltransferases may alter plant immunity in both NPR1-dependent and NPR1-independent ways (Jin et al., 2018). Under normal conditions, a small fraction of monomeric NPR1 is present inside the nucleus and interacts with TGA and HAC to form a ternary HAC–NPR1–TGA complex (Jin et al., 2018). However, some TGAs that are not part of this complex binds to *PR* promoters and inhibit *PR* transcription. Thus, HAC–NPR1–TGA complex is not recruited to *PR* chromatin in this situation. During pathogen attack, SA upsurges and binds to NPR1 in the nucleus. HAC1, HAC5, and NPR1 create a coactivator complex and bind to *PR* chromatin *via* TGAs, forming HAC–NPR1–TGA complex to promote *PR* transcription through histone acetylation-mediated epigenetic reprogramming (Jin et al., 2018). Mutation of *hac1/5* leads to reduced pathogen-induced expression of various SA biosynthesis or accretion-related genes like *EDS5*, *PAD4*, and *ICS1*, whereas *npr1* mutation did not affect the induction of these genes in *hac1/5* mutants (Jin et al., 2018). This indicates that HACs also regulate SA biosynthesis or accumulation-related genes in an NPR1-independent manner (Jin et al., 2018).

Even though HAC1/5 may not be essential for NPR1 to interact with free TGAs, they are likely required for NPR1 to bind to TGAs efficiently in the chromatin. One possibility is that acetylation of histones by HAC, which was recruited *via* NPR1 to *PR* gene chromatin, can alter the local conformation of chromatin so that the HAC–NPR1–TGA complex is more stable in connection with chromatin (Jin et al., 2018). Conversely, HAC could serve as an adapter, creating multivalent associations with transcription factors, thereby maintaining NPR1’s engagement with *PR* chromatin (Jin et al., 2018). Another possibility is that SA-binding to NPR1 may modify the HAC-NPR1 complex or the ternary HAC–NPR1–TGA complex, allowing for more efficient DNA binding of TGAs on *PR* promoters (Jin et al., 2018). Hence, HAC may help enhance or stabilize the formation of the HAC–NPR1–TGA complex on *PR* chromatin and facilitate the transcription of *PR* genes during plant defense responses.

## NPR1: A Salicylic Acid Receptor and a Master Regulator

### Dynamic structural changes determine the transcriptional regulation efficiency of NPR1

Several studies have identified SA-regulated downstream signaling components. Among them, NPR1 is considered to be a vital SA receptor and a leading redox controller of SA-regulated defense signaling by modulating the expression of a series of disease-resistant genes. Of the approximate 2,800 SA-responsive genes, the great majority (more than 98%) are NPR1-dependent. Structurally, NPR1 contains several characterized functional elements, including a BTB/POZ domain in the N-terminus, ankyrin repeat and transactivation domains in the C-terminus, and a nuclear localization sequence, with the BTB/POZ sequence showing resemblance to E3 ligase adaptor motif (Dong, 2004; Dieterle et al., 2005; Rochon et al., 2006). Interestingly, the paralogs of NPR1, NPR3, and NPR4, have been verified to possess an E3 ligase adaptor domain and were identified as Cullin 3 RING E3 ligase adaptors, which promoted the degradation of NPR1 in the nucleus (Spoel et al., 2009; Fu et al., 2012). The studies on *npr1* mutant plants demonstrated that malfunction of NPR1 completely abolishes plant defense against pathogens due to interruption in SA-regulated downstream signaling and transcription.

During pathogen infection, SA directly binds to NPR1 to regulate its activity and stability, which is essential for its downstream activation of effector proteins/genes. Upon activation, NPR1 undergoes several post-translation modifications. In the normal state, NPR1 is resting in the form of high-molecular-weight oligomers in the cytoplasm (Withers and Dong, 2016). Under pathogenic attacks, the presence of a higher concentration of SA disturbs redox balance in the cytoplasm, changing NPR1 from oligomers to monomers *via* thioredoxin (TRX-h3 and TRX-h5)-mediated reduction of a cysteine residue (Cys156) in NPR1 (Waszczak et al., 2015; Withers and Dong, 2016). In normal conditions, NPR1 is associated with transcription repressor and is phosphorylated at serine residues 55 and 59, thus blocking its promotion of the expression of SA-responsive genes (Waszczak et al., 2015; Withers and Dong, 2016). Upon pathogenic infection, increasing cellular concentration of SA leads to the dephosphorylation of Ser55/Ser59 and SUMOylation at the SUMO-interacting motif 3, which triggers the phosphorylation of Ser11/Ser15, in NPR1 (Saleh et al., 2015). The recent findings of Zavaliev et al. (2020) demonstrated that dephosphorylation Ser55/Ser59 and phosphorylation of Ser11/Ser15 facilitate NPR1 to enter the nucleus or assemble cell death regulators and stress response proteins to form punctate structures known as SA-induced NPR1 condensates (SINCs). The authors further demonstrated that SA also promotes NPR1’s interaction with Cullin3 (CUL3) E3 ligase by phosphorylating NPR1 at Ser11/Ser15 to stimulate NPR1 turnover (Zavaliev et al., 2020).

NPR1 ubiquitination by CUL3 and degradation by the 26 proteasome in the nucleus also influence SINC formation in the cytoplasm, as demonstrated in the SUMOylation-deficient mutant of NPR1 (Zavaliev et al., 2020). In SNICs, NPR1 enables SA-responsive ubiquitination of target proteins to boost cell survival; therefore, SINCs serve as a site for recruitment and ubiquitination (with the help of CUL3 E3 ligases) of key members of the stress response machinery, such as EDS1 and WRKY54/70, to promote cell survival. This is because, under pathogen infection, SA level increases, which inhibits the CUL3^NPR4^ but promotes CUL3^NPR3^-mediated degradation of NPR1, leading to ETI. However, in adjacent cells, a lower concentration of SA is not sufficient to promote the interaction of NPR3 with NPR1, which enables the accumulation of NPR1 to suppress cell death. NPR1-mediated SINC formation may be essential for robust transcriptional reprogramming to redirect energy for defense instead of growth upon pathogenic infection (Peng et al., 2021; Chen et al., 2021b). However, the mechanism through which SA triggers the dynamic formation of SINCs requires future elucidation.

### NPR1-Mediated Transcriptional Regulation of Plant Immunity

The NPR1 protein does not contain a canonical DNA-binding domain; instead, after monomerization and re-localization to the nucleus, NPR1 promotes transcriptional activation by interacting with appropriate transcription factors to mediate the expression of more than 2,000 genes (Liu et al., 2018).

In *Arabidopsis*, NPR1 interacts with seven out of a total of 10 TGA TFs. TGA1 and TGA4 interact with NPR1 in a redox-dependent manner, while TGA2, TGA3, TGA5, and TGA6 are prerequisites for SA-regulated gene expression (Lindermayr et al., 2010; Spoel and Loake, 2011; Herrera-Vásquez et al., 2015). It has been established that SA induces reduction of the disulfide bridges in TGA proteins, which enable them to interact with NPR1. In turn, TGA and NPR1 interaction activate the expression of *PR1* (Fan and Dong, 2002; Rochon et al., 2006).

Besides TGAs, TCP and WRKY TFs have also been implicated in SA-mediated SAR responses. A recent study demonstrated that TCP8, TCP14, and TCP15 physically interacted with NPR1, and TCP15 binds to the promoter of *PR5* under the influence of NPR1 (Li et al., 2018). However, the precise molecular mechanism of NPR1-assisted TCP binding to the promoter of *PR5* is obscure, which warrants further investigation (Li et al., 2018). The presence of several WRKY TF binding sites (W-box elements) in the promoter region of *NPR1* implies that NPR1 may be cross-regulated by WRKY TFs (Pajerowska-Mukhtar et al., 2013). A growing body of evidence suggests that the interaction of WRKY TFs with NPR1 stimulates the expression of SA-responsive genes (Pajerowska-Mukhtar et al., 2013), and the expression of these genes is associated with the strengthening of *R* gene-dependent resistance (Meena and Swapnil, 2019). However, despite the finding of three WRKY TF binding sites, that is, W-box (TTGAC) elements in *NPR1* promoter, the precise molecular mechanism underlying the regulation of NPR1 expression and function by WRKY TFs remains to be clarified. One useful clue for further research is that NPR1 could promote its own expression by binding to self-promoter through interacting with WRKY18 (Chen et al., 2019).

## EDS1: A Multitalented Defender

### EDS1 and Its Interacting Partners Trigger Distinct Pathways

One important mechanism underpinning SA’s involvement in plant immunity (PTI, ETI, and SAR) is transcriptional reprogramming of SA biosynthesis genes (Wildermuth et al., 2001). The downstream responses of SA-mediated immunity are modulated by the nucleocytoplasmic regulator NPR1, which is a transcriptional coactivator of SA-dependent local and systemic immunity (Fu and Dong, 2013; Saleem et al., 2021). On the other hand, EDS1 is a necessary component in both basal and R protein-mediated resistance (TIR-NBS-LRR class) against virulent and avirulent pathogens (Peart et al., 2002; Li et al., 2019a). In flowering plants, EDS1 forms functional heterodimers with SAG101 or PAD4, with EDS1–SAG101 and EDS1–PAD4 heterodimers having diverse functions in plant immunity (Wiermer et al., 2005; Wagner et al., 2013). The interaction of these regulatory nodes distinctly reprograms transcriptional activities in infected cells and initiates the production of SA and other stress signals to limit the growth of invading pathogens. EDS1 and PAD4 are crucial for regulating plant basal immunity. The heterodimer complex of EDS1 and PAD4 stimulates SA accumulation, which in turn induces the expression of *EDS1* and *PAD4*. As a result, they are forming a positive feedback loop to enhance SA-activated immune system (Jirage et al., 1999; Feys et al., 2001; Vlot et al., 2009). Usually, EDS1 and PAD4 work together, but they can also function independently. For example, EDS1 interacts with SAG101–NRG1 module in TNL-triggered ETI to induce host cell death and transcriptional reprogramming without needing PAD4 (Lapin et al., 2019).

EDS1–PAD4 interacts with ADR1 type of RNLs to regulate basal immunity by transcriptionally modifying SA signaling pathway to induce local and systemic defense under TNL/CNL-triggered ETI (Bonardi et al., 2011; Cui et al., 2017; Lapin et al., 2019; Saile et al., 2020). Recently, Sun et al. (2021) showed that EDS1–PAD4–ADR1 and EDS1–SAG101–NRG1 constitute two separate immunity signaling nodes downstream of NLR activation to boost basal immunity against pathogens.

### Nucleocytoplasmic Distribution of EDS1 During Plant Innate Immune Responses

The shuttling of EDS1 and PAD4 in the cytoplasm and the nucleus is important for defense activation processes (Cheng et al., 2009; García et al., 2010). Most of the regulatory proteins are present in the cytoplasm; however, EDS1–SAG101 heterodimer exists mainly in the nucleus. The EDS1-PAD4 heterodimer is present in both the cytoplasm and the nucleus, whereas the complex of EDS1, PAD4, and SAG101 is predominantly nuclear-localized. Less is known about the mechanism that maintains a delicate balance of these regulatory proteins in the cytosolic and nuclear compartments. Interestingly, recent molecular studies highlighted the roles of EIJ1 and RIN13 in regulating the subcellular distributions of EDS1 or PAD4 in infected cells. EIJ1, a DnaJ type of chaperone, rapidly relocalizes from the chloroplast to the cytoplasm, where it interacts with EDS1, during the early stage of pathogen infection in *Arabidopsis* (Liu et al., 2021). This interaction prevents EDS1 trafficking to the nucleus and prohibits the elicitation of unnecessary immune responses to short-term pathogenic stimulation. However, when plants are under prolonged attack of pathogens, EIJ1–EDS1 complex degrades cytoplasmic EDS1, and the accumulation of EDS1 increases in the nucleus to reinforce long-term resistance in the plants (Liu et al., 2021). Similarly, RIN13 could drive PAD4 into the nucleus (Liu et al., 2021). The shuttling of EDS1 and PAD4 proteins from the cytoplasm to the nucleus is necessary to activate defense gene expression and for the accumulation of stress signaling molecules, such as SA. These findings point out the vital roles of EIJ1 and RIN13 during pathogen invasion and provide new information about how the subcellular localization of EDS1/PAD4 is regulated to confer resistance in pathogen-challenged plants (Liu et al., 2021).

### EDS1-Mediated SA-Dependent/Independent Signaling

Enhanced Disease Susceptibility 1 initiates SA-dependent and SA-independent pathways to transcriptionally reprogram infected cells for immunity and localized cell death (Bartsch et al., 2006; Cui et al., 2017). The existence of multiple pathways warrants robust activation of defense responses. If one pathway is blocked due to manipulation by pathogen effector or other unknown reasons, the alternate pathway would still ensure defense (Cui et al., 2017). Overexpression analysis of EDS1 and PAD4 validates the expression of both SA-dependent and SA-independent genes. Both SA-dependent and SA-independent functions of EDS1/PAD4 mediate plant basal immunity and ETI (Cui et al., 2017). The SA-dependent pathway is associated with pathogen-induced SA accumulation to boost resistance, while the other one is independent of SA synthesis *via* ICS1 by recruiting other functional partners (ALD1/FMO1-dependent) to amplify resistance (Bartsch et al., 2006; Cui et al., 2017). In the SA-dependent pathway, EDS1 heterodimer promotes SA biosynthesis *via* ICS1 and transcriptionally induces defense responses. In the absence of EDS1, heterodimer partners like PAD4 and SGS101 are ineffective in promoting plant defense due to improper accumulation of SA and *PR1* expression (Rietz et al., 2011).

Enhanced Disease Susceptibility 1 regulates the SA-independent signaling cascade by triggering the transcriptional activation of FMO1, irrespective of local SA production/accumulation (Bartsch et al., 2006; Cui et al., 2017). The SA-independent branch of EDS1 with PAD4 module activates FMO1 and induces *PR1* gene expression due to enhanced accumulation of free and conjugated SA, but this increase in SA was not associated with ICS1 activity under pathogenic attack. Analysis of *eds1-2* and *pad4-1* single and double mutants revealed that pathogen effector-induced *FMO1* expression was significantly reduced, which clearly suggests that the activation of FMO1 and SA accumulation is due to functions of the EDS1–PAD4 complex (Joglekar et al., 2018). Additionally, it was observed that EDS1-induced SA-independent immunity was effective against the infections by *Pst* DC3000 and the oomycete pathogen *H. arabidopsidis* in *Arabidopsis*, illustrating that the mobilization of SA-independent defense pathways by EDS1*/*PAD4 signaling is an effective immune response in controlling pathogenic diseases in plants (Mishina and Zeier, 2006; Joglekar et al., 2018). Likewise, when *Arabidopsis* plants were infected with the *Pst* carrying *avrRpt2*, EDS1-meditated SA-independent contribution to defense responses appeared stronger with sustained MAPK activation (Hartmann et al., 2018; Wang et al., 2018). Lastly, EDS1-PAD4 also controls the receptor-like kinase BAK1-mediated cell death signaling in an SA-independent manner because cell death of *bak1-3 bkk1-1 sid2-3* in *eds1* or *pad4* background was suppressed, which suggests that EDS1 contributes to BAK1-mediated cell death pathway *via* SA-independent signaling pathway (Gao et al., 2017). Clearly, the SA-independent branch of EDS1 signaling is active in the stimulation of local immunity in infected plants (Hartmann et al., 2018; Wang et al., 2018).

### EDS1 Crosstalking With Other Regulatory Hubs

EDS1 suppresses the function of JA regulators to reinforce SA-mediated plant defense. JA signaling under pathogenic infection works antagonistically to SA signaling (Vlot et al., 2009; Yang et al., 2012). *Pst* DC3000 toxin coronatine (COR), a bacterial JA mimicker, could disable SA signaling *via* modulation of JA signaling pathways (Brooks et al., 2005; Kazan and Lyons, 2014; Yang et al., 2017). Upon the inoculation of *Pst* DC3000 carrying *avrRps4*, EDS1/PAD4 complexes mobilize a major portion of the TNL (RRS1-S*/*RPS4) immune response to counter bacterial COR-mediated MYC2 transcriptional reprogramming of JA responsive genes (*VSP1* and *JAZ10*). Molecular analysis shows that EDS1 antagonizes MYC2 function in the nucleus rather than its entry into the nucleus *via* suppressing MYC2 binding to a responsive promoter (*pANAC019*) and improving the SA defense sector independent of EDS1-triggered SA synthesis (Cui et al., 2018). Similarly, gibberellic acid repressors, DELLA proteins, act as modulators between growth and resistance responses under pathogenic infection. When plants are infected, EDS1 rapidly induces and promotes SA-induced defense responses. At the same time, defense signaling activates EDS1-dependent DELLA stabilization to suppress plant growth. Later, the stabilized DELLAs interact with EDS1 to slow down SA production and repress resistance response to maintain the balance between growth and defense under long-term pathogen attack. This suggests that regulatory feedback exists between EDS1-DELLA and SA under pathogenic infections (Li et al., 2019b). Recently, it was also found that EDS1 interacts with BRASSINAZOLE RESISTANT 1 (BZR1), a major regulator of BR-induced transcriptional changes, to regulate immune responses in infected *Arabidopsis* plants (Qi et al., 2021). During compatible pathogen infection, EDS1 negatively regulates BZR1 signaling by binding to BZR1, which suppresses the expression of BR-responsive genes (e.g., *EXP8* and *SAUR15*) and BR-promoted growth with concomitant onset of efficient PTI. On the other hand, presence of sufficient BZR1 in the cytoplasm is required for effective induction of RPS4-mediated ETI *via* facilitating the dissociation of EDS1 and RPS4 dimers in the cytoplasm, which is a crucial step in the ETI controlled by RPS4 (Qi et al., 2021). Thus, it seems that extensive crosstalking exists among EDS1 and diverse regulatory hubs (e.g., EDS1, BZR1, MYC2, and DELLA), which contributes to the mounting of effective and yet balanced disease resistance in different pathogen–host interactions.

## NPR1 and EDS1: Two Interdependent and Synergistic Coactivators

Both EDS1 and NPR1 function as central hubs in plant immunity, and they are both targeted by pathogen effectors (Chen et al., 2021a). Through yeast two-hybrid screening, EDS1 was identified as a NPR1-interacting protein. Importantly, Chen et al. (2021a) demonstrated that EDS1 has transcriptional activation activity. Through analyzing EDS1 deletions and truncations, Chen et al. (2021a) found that two regions in EDS1 are necessary and sufficient for EDS1’s transcriptional activation activity. Amino acid sequence analysis revealed that acidic and hydrophobic amino acids are enriched in these two regions, which are presumably involved in the ionic and hydrophobic interactions with their target molecules. Therefore, EDS1 harbors two acidic transcriptional activation domains, similar to those identified in the TFs, such as P53, GCN4, GAL4, and VP16 (Chen et al., 2021a). This realization provides new insight into EDS1’s function in the regulation of downstream defense genes upon SA accumulation under pathogenic attack (Chen et al., 2021a).

Further examination revealed that EDS1 and NPR1 bind to similar regions in the *PR1* promoter, which are TGA-binding *as-1* and WRKY-binding W-box elements (Chen et al., 2021a). EDS1 and NPR1 synergistically promote the expression of *PR* genes. Another transcriptional coactivator, CDK8, physically interacts with NPR1 and EDS1 and acts as a bridge between transcription factors and RNA polymerase II to promote the expression of plant defense genes (Chen et al., 2019). NPR1 facilitates SA-induced EDS1 chromatin binding and *PR1* activation by effectively recruiting EDS1 to the *PR1* promoter (Chen et al., 2021a; Figure 1). Thus, physical interaction between EDS1 and NPR1 plays an intrinsic role in the interaction of EDS1 with *PR1* promoter, while the two transcriptional coactivators, EDS1 and NPR1, may directly recruit the Mediator complex in the transcription machinery to reinforce the expression of SA-responsive genes and thus SA-mediated defense responses (Chen et al., 2021a).

Genetic experiment revealed that NPR1 transcriptionally upregulates *EDS1* expression *via* TGA2-NPR1 interaction, and *in planta* analysis discovered that EDS1 stabilizes NPR1 protein level by preventing its degradation to sustain immune responses under pathogenic infection (Chen et al., 2021a). These results support the idea that the functions of EDS1 and NPR1 in SA-mediated immunity are interdependent.

## Conclusion and Future Prospects

In the past two decades, tremendous progress has been made toward understanding SA signaling and regulation under pathogenic attack in plants. However, many questions still need to be answered. As transcriptional coactivators, plant Mediator subunits play vital roles in the transcriptional regulations of plant immunity, but this driving force in SA-mediated plant defense has yet to be fully understood. For instance, the functions of MED11, MED22, MED26, and other subunits in SA signaling still need to be explored. The MED12 and MED13 subunits of the CDK8 module are involved in the transcriptional regulation of *NPR1* and its target genes, but the transcriptional factors that interact with these two subunits are still unknown. Similarly, the TFs that interact with MED5/14/15/16/19, which are involved in SA signaling, remain to be identified.

The precise biochemical roles of EDS1, NPR1, and related components in signaling PTI and ETI need further elucidation (Bjornson and Zipfel, 2021; Yuan et al., 2021). For instance, how are Ca^2+^ signatures and Ca^2+^ channels integrated with activation of the transcriptional coactivators, such as NPR1 and EDS1, to orchestrate different defense responses in both PTI and ETI immune systems? What genes do EDS1 and NPR1 control during these processes? More detailed genetic and biochemical investigations of the spatial and temporal regulation of defense genes regulated by EDS1 and NPR1 will help to better understand how EDS1 and NPR1 control plant immunity.

As a transcriptional coactivator without a DNA-binding domain, EDS1 has to interact with appropriate TFs, such as TGAs and WRKYs, to facilitate transcription. Therefore, it is necessary to identify the TFs that interact with EDS1 in different pathosystems. Similarly, further studies of additional transcriptional regulators, including the HACs, HDAs, and other epigenetic regulators that directly or indirectly interact with EDS1-NPR1-CDK8 complex, are needed to facilitate an in-depth understanding of coactivator-mediated plant immunity. Previous studies have revealed the crystal structure of truncated NPR4, EDS1/PAD4, and EDS1/SAG101 heterodimers, but the crystal structure of EDS1-NPR1-CDK8 complex remains undetermined. In view of the crucial importance of EDS1-NPR1-CDK8 in SA-mediated immunity (Chen et al., 2021a), it now becomes necessary to determine the crystal structure of this complex, the insight from which will guide further and deeper functional and mechanistic studies of transcriptional coactivators in controlling plant immunity.

## Author Contributions

MK drew the figures. All authors contributed to writing the article and approved the submitted version.

## Funding

This work is supported by the grant from National Science Foundation (IOS-1758994) to ZF and by grants from Jiangsu University High-Level Talent Funding (20JDG34), Natural Science Foundation of Jiangsu Province (BK20211319), and National Natural Science Foundation of China (32000201) to JC.

## Conflict of Interest

The authors declare that the research was conducted in the absence of any commercial or financial relationships that could be construed as a potential conflict of interest.

## Publisher’s Note

All claims expressed in this article are solely those of the authors and do not necessarily represent those of their affiliated organizations, or those of the publisher, the editors and the reviewers. Any product that may be evaluated in this article, or claim that may be made by its manufacturer, is not guaranteed or endorsed by the publisher.

## References

[ref2] Bartsch M. Gobbato E. Bednarek P. Debey S. Schultze J. L. Bautor J.. (2006). Salicylic acid–independent ENHANCED DISEASE SUSCEPTIBILITY1 signaling in Arabidopsis immunity and cell death is regulated by the monooxygenase FMO1 and the nudix hydrolase NUDT7. Plant Cell 18, 1038–1051. doi: 10.1105/tpc.105.039982, PMID: 16531493 PMC1425861

[ref4] Betsuyaku S. Katou S. Takebayashi Y. Sakakibara H. Nomura N. Fukuda H. (2018). Salicylic acid and jasmonic acid pathways are activated in spatially different domains around the infection site during effector-triggered immunity in *Arabidopsis thaliana*. Plant Cell Physiol. 59, 8–16. doi: 10.1093/pcp/pcx181, PMID: 29177423 PMC6012717

[ref5] Bjornson M. Zipfel C. (2021). Plant immunity: crosstalk between plant immune receptors. Curr. Biol. 31, R796–R798. doi: 10.1016/j.cub.2021.04.080, PMID: 34157265

[ref6] Bonardi V. Tang S. Stallmann A. Roberts M. Cherkis K. Dangl J. L. (2011). Expanded functions for a family of plant intracellular immune receptors beyond specific recognition of pathogen effectors. PNAS 108, 16463–16468. doi: 10.1073/pnas.1113726108, PMID: 21911370 PMC3182704

[ref7] Bonawitz N. D. Soltau W. L. Blatchley M. R. Powers B. L. Hurlock A. K. Seals L. A.. (2012). REF4 and RFR1, subunits of the transcriptional coregulatory complex mediator, are required for phenylpropanoid homeostasis in Arabidopsis. J. Biol. Chem. 287, 5434–5445. doi: 10.1074/jbc.M111.312298, PMID: 22167189 PMC3285322

[ref8] Brooks D. M. Bender C. L. Kunkel B. N. (2005). The *Pseudomonas syringae* phytotoxin coronatine promotes virulence by overcoming salicylic acid-dependent defences in *Arabidopsis thaliana*. Mol. Plant Pathol. 6, 629–639. doi: 10.1111/j.1364-3703.2005.00311.x, PMID: 20565685

[ref9] Caillaud M.-C. Asai S. Rallapalli G. Piquerez S. Fabro G. Jones J. D. (2013). A downy mildew effector attenuates salicylic acid–triggered immunity in Arabidopsis by interacting with the host mediator complex. PLoS Biol. 11:e1001732. doi: 10.1371/journal.pbio.1001732, PMID: 24339748 PMC3858237

[ref10] Canet J. V. Dobón A. Tornero P. (2012). Non-recognition-of-BTH4, an Arabidopsis mediator subunit homolog, is necessary for development and response to salicylic acid. Plant Cell 24, 4220–4235. doi: 10.1105/tpc.112.103028, PMID: 23064321 PMC3517246

[ref11] Chen H. Chen J. Li M. Chang M. Xu K. Shang Z.. (2017). A bacterial type III effector targets the master regulator of salicylic acid signaling, NPR1, to subvert plant immunity. Cell Host Microbe 22, 777–788.e7. doi: 10.1016/j.chom.2017.10.019, PMID: 29174403

[ref12] Chen J. Clinton M. Qi G. Wang D. Liu F. Fu Z. Q. (2020). Reprogramming and remodeling: transcriptional and epigenetic regulation of salicylic acid-mediated plant defense. J. Exp. Bot. 71, 5256–5268. doi: 10.1093/jxb/eraa072, PMID: 32060527

[ref13] Chen J. Li M. Liu L. Chen G. Fu Z. Q. (2021c). ZAR1 resistosome and helper NLRs: bringing in calcium and inducing cell death. Mol. Plant 14, 1234–1236. doi: 10.1016/j.molp.2021.06.026, PMID: 34198009

[ref14] Chen H. Li M. Qi G. Zhao M. Liu L. Zhang J.. (2021a). Two interacting transcriptional coactivators cooperatively control plant immune responses. Sci. Adv. 7:eabl7173. doi: 10.1126/sciadv.abl7173, PMID: 34739308 PMC8570602

[ref15] Chen J. Mohan R. Zhang Y. Li M. Chen H. Palmer I. A.. (2019). NPR1 promotes its own and target gene expression in plant defense by recruiting CDK8. Plant Physiol. 181, 289–304. doi: 10.1104/pp.19.00124, PMID: 31110139 PMC6716257

[ref16] Chen J. Zhang J. Kong M. Freeman A. Chen H. Liu F. (2021b). More stories to tell: NONEXPRESSOR OF PATHOGENESIS-RELATED GENES1, a salicylic acid receptor. Plant Cell Environ. 44, 1716–1727. doi: 10.1111/pce.14003, PMID: 33495996

[ref17] Cheng Y. T. Germain H. Wiermer M. Bi D. Xu F. García A. V.. (2009). Nuclear pore complex component MOS7/Nup88 is required for innate immunity and nuclear accumulation of defense regulators in Arabidopsis. Plant Cell 21, 2503–2516. doi: 10.1105/tpc.108.064519, PMID: 19700630 PMC2751965

[ref18] Cui H. Gobbato E. Kracher B. Qiu J. Bautor J. Parker J. E. (2017). A core function of EDS1 with PAD4 is to protect the salicylic acid defense sector in Arabidopsis immunity. New Phytol. 213, 1802–1817. doi: 10.1111/nph.14302, PMID: 27861989

[ref19] Cui H. Qiu J. Zhou Y. Bhandari D. D. Zhao C. Bautor J.. (2018). Antagonism of transcription factor MYC2 by EDS1/PAD4 complexes bolsters salicylic acid defense in Arabidopsis effector-triggered immunity. Mol. Plant 11, 1053–1066. doi: 10.1016/j.molp.2018.05.007, PMID: 29842929

[ref20] Delaney T. P. Uknes S. Vernooij B. Friedrich L. Weymann K. Negrotto D.. (1994). A central role of salicylic acid in plant disease resistance. Science 266, 1247–1250. doi: 10.1126/science.266.5188.1247 17810266

[ref21] Devadas S. K. Raina R. (2002). Preexisting systemic acquired resistance suppresses hypersensitive response-associated cell death in Arabidopsis hrl1 mutant. Plant Physiol. 128, 1234–1244. doi: 10.1104/pp.010941, PMID: 11950972 PMC154251

[ref23] Dieterle M. Thomann A. Renou J. P. Parmentier Y. Cognat V. Lemonnier G.. (2005). Molecular and functional characterization of Arabidopsis Cullin 3A. Plant J. 41, 386–399. doi: 10.1111/j.1365-313X.2004.02302.x, PMID: 15659098

[ref24] Ding Y. Sun T. Ao K. Peng Y. Zhang Y. Li X.. (2018). Opposite roles of salicylic acid receptors NPR1 and NPR3/NPR4 in transcriptional regulation of plant immunity. Cell 173, 1454–1467.e15. doi: 10.1016/j.cell.2018.03.044, PMID: 29656896

[ref25] Dong X. (2004). NPR1, all things considered. Curr. Opin. Plant Biol. 7, 547–552. doi: 10.1016/j.pbi.2004.07.005, PMID: 15337097

[ref26] Fan W. Dong X. (2002). *In vivo* interaction between NPR1 and transcription factor TGA2 leads to salicylic acid–mediated gene activation in Arabidopsis. Plant Cell 14, 1377–1389. doi: 10.1105/tpc.001628, PMID: 12084833 PMC150786

[ref27] Feys B. J. Moisan L. J. Newman M. A. Parker J. E. (2001). Direct interaction between the Arabidopsis disease resistance signaling proteins, EDS1 and PAD4. EMBO J. 20, 5400–5411. doi: 10.1093/emboj/20.19.5400, PMID: 11574472 PMC125652

[ref28] Fu Z. Q. Dong X. J. (2013). Systemic acquired resistance: turning local infection into global defense. Annu. Rev. Plant Biol. 64, 839–863. doi: 10.1146/annurev-arplant-042811-105606, PMID: 23373699

[ref29] Fu Z. Q. Yan S. Saleh A. Wang W. Ruble J. Oka N.. (2012). NPR3 and NPR4 are receptors for the immune signal salicylic acid in plants. Nature 486, 228–232. doi: 10.1038/nature11162, PMID: 22699612 PMC3376392

[ref30] Gao Y. Wu Y. Du J. Zhan Y. Sun D. Zhao J.. (2017). Both light-induced SA accumulation and ETI mediators contribute to the cell death regulated by BAK1 and BKK1. Front. Plant Sci. 8:622. doi: 10.3389/fpls.2017.00622, PMID: 28487714 PMC5403931

[ref31] García A. V. Blanvillain-Baufumé S. Huibers R. P. Wiermer M. Li G. Gobbato E.. (2010). Balanced nuclear and cytoplasmic activities of EDS1 are required for a complete plant innate immune response. PLoS Pathog. 6:e1000970. doi: 10.1371/journal.ppat.1000970, PMID: 20617163 PMC2895645

[ref32] Guo J. Wei L. Chen S. S. Cai X. W. Su Y. N. Li L.. (2021). The CBP/p300 histone acetyltransferases function as plant-specific MEDIATOR subunits in Arabidopsis. J. Integr. Plant Biol. 63, 755–771. doi: 10.1111/jipb.13052, PMID: 33325122

[ref33] Hartmann M. Zeier T. Bernsdorff F. Reichel-Deland V. Kim D. Hohmann M.. (2018). Flavin monooxygenase-generated *N*-hydroxypipecolic acid is a critical element of plant systemic immunity. Cell 173, 456–469.e16. doi: 10.1016/j.cell.2018.02.049, PMID: 29576453

[ref35] Herrera-Vásquez A. Carvallo L. Blanco F. Tobar M. Villarroel-Candia E. Vicente-Carbajosa J.. (2015). Transcriptional control of glutaredoxin GRXC9 expression by a salicylic acid-dependent and NPR1-independent pathway in Arabidopsis. Plant Mol. Biol. Rep. 33, 624–637. doi: 10.1007/s11105-014-0782-5, PMID: 26696694 PMC4677692

[ref36] Hu Y. Lu Y. Zhao Y. Zhou D.-X. (2019). Histone acetylation dynamics integrates metabolic activity to regulate plant response to stress. Front. Plant Sci. 10:1236. doi: 10.3389/fpls.2019.01236, PMID: 31636650 PMC6788390

[ref37] Huang J. Sun Y. Orduna A. R. Jetter R. Li X. (2019). The mediator kinase module serves as a positive regulator of salicylic acid accumulation and systemic acquired resistance. Plant J. 98, 842–852. doi: 10.1111/tpj.14278, PMID: 30739357

[ref38] Jin H. Choi S.-M. Kang M.-J. Yun S.-H. Kwon D.-J. Noh Y.-S.. (2018). Salicylic acid-induced transcriptional reprogramming by the HAC–NPR1–TGA histone acetyltransferase complex in Arabidopsis. Nucleic Acids Res. 46, 11712–11725. doi: 10.1093/nar/gky847, PMID: 30239885 PMC6294559

[ref39] Jirage D. Tootle T. L. Reuber T. L. Frost L. N. Feys B. J. Parker J. E.. (1999). *Arabidopsis thaliana* PAD4 encodes a lipase-like gene that is important for salicylic acid signaling. PNAS 96, 13583–13588. doi: 10.1073/pnas.96.23.13583, PMID: 10557364 PMC23991

[ref40] Joglekar S. Suliman M. Bartsch M. Halder V. Maintz J. Bautor J.. (2018). Chemical activation of EDS1/PAD4 signaling leading to pathogen resistance in Arabidopsis. Plant Cell Physiol. 59, 1592–1607. doi: 10.1093/pcp/pcy106, PMID: 29931201

[ref41] Jones J. D. Vance R. E. Dangl J. L. (2016). Intracellular innate immune surveillance devices in plants and animals. Science 354:aaf6395. doi: 10.1126/science.aaf6395, PMID: 27934708

[ref42] Kazan K. Lyons R. (2014). Intervention of phytohormone pathways by pathogen effectors. Plant Cell 26, 2285–2309. doi: 10.1105/tpc.114.125419, PMID: 24920334 PMC4114936

[ref44] Kumar V. Thakur J. K. Prasad M. (2021). Histone acetylation dynamics regulating plant development and stress responses. Cell. Mol. Life Sci. 78, 4467–4486. doi: 10.1007/s00018-021-03794-x, PMID: 33638653 PMC11072255

[ref45] Lai F. Orom U. A. Cesaroni M. Beringer M. Taatjes D. J. Blobel G. A.. (2013). Activating RNAs associate with mediator to enhance chromatin architecture and transcription. Nature 494, 497–501. doi: 10.1038/nature11884, PMID: 23417068 PMC4109059

[ref46] Lapin D. Kovacova V. Sun X. Dongus J. A. Bhandari D. Von Born P.. (2019). A coevolved EDS1-SAG101-NRG1 module mediates cell death signaling by TIR-domain immune receptors. Plant Cell 31, 2430–2455. doi: 10.1093/plcell/koab085, PMID: 31311833 PMC6790079

[ref47] Larivière L. Seizl M. Cramer P. (2012). A structural perspective on mediator function. Curr. Opin. Cell Biol. 24, 305–313. doi: 10.1016/j.ceb.2012.01.007, PMID: 22341791

[ref48] Li M. Chen H. Chen J. Chang M. Palmer I. A. Gassmann W.. (2018). TCP transcription factors interact with NPR1 and contribute redundantly to systemic acquired resistance. Front. Plant Sci. 9:1153. doi: 10.3389/fpls.2018.01153, PMID: 30154809 PMC6102491

[ref49] Li Y. Yang Y. Hu Y. Liu H. He M. Yang Z.. (2019b). DELLA and EDS1 form a feedback regulatory module to fine-tune plant growth–defense tradeoff in Arabidopsis. Mol. Plant 12, 1485–1498. doi: 10.1016/j.molp.2019.07.006, PMID: 31382023

[ref50] Li S. Zhao J. Zhai Y. Yuan Q. Zhang H. Wu X.. (2019a). The hypersensitive induced reaction 3 (HIR 3) gene contributes to plant basal resistance *via* an EDS 1 and salicylic acid-dependent pathway. Plant J. 98, 783–797. doi: 10.1111/tpj.14271, PMID: 30730076

[ref51] Lindermayr C. Sell S. Müller B. Leister D. Durner J. J. (2010). Redox regulation of the NPR1-TGA1 system of *Arabidopsis thaliana* by nitric oxide. Plant Cell 22, 2894–2907. doi: 10.1105/tpc.109.066464, PMID: 20716698 PMC2947166

[ref52] Liu C. Atanasov K. E. Arafaty N. Murillo E. Tiburcio A. F. Zeier J.. (2020a). Putrescine elicits ROS-dependent activation of the salicylic acid pathway in *Arabidopsis thaliana*. Plant Cell Environ. 43, 2755–2768. doi: 10.1111/pce.13874 32839979

[ref53] Liu Q. Bischof S. Harris C. J. Zhong Z. Zhan L. Nguyen C.. (2020b). The characterization of mediator 12 and 13 as conditional positive gene regulators in Arabidopsis. Nat. Commun. 11, 2798–2713. doi: 10.1038/s41467-020-16651-5, PMID: 32493925 PMC7271234

[ref54] Liu H. Li Y. Hu Y. Yang Y. Zhang W. He M.. (2021). EDS1-interacting J protein 1 (EIJ1) is an essential negative regulator of plant innate immunity in Arabidopsis. Plant Cell 33, 153–171. doi: 10.1093/plcell/koaa007, PMID: 33751092 PMC8136891

[ref55] Liu F. Li X. Wang M. Wen J. Yi B. Shen J.. (2018). Interactions of WRKY 15 and WRKY 33 transcription factors and their roles in the resistance of oilseed rape to Sclerotinia infection. Plant Biotechnol. J. 16, 911–925. doi: 10.1111/pbi.12838, PMID: 28929638 PMC5867032

[ref57] Malik N. Ranjan R. Parida S. K. Agarwal P. Tyagi A. K. (2020). Mediator subunit OsMED14_1 plays an important role in rice development. Plant J. 101, 1411–1429. doi: 10.1111/tpj.14605, PMID: 31702850

[ref58] Malik S. Roeder R. G. (2010). The metazoan mediator co-activator complex as an integrative hub for transcriptional regulation. Nat. Rev. Genet. 11, 761–772. doi: 10.1038/nrg2901, PMID: 20940737 PMC3217725

[ref59] Mao X. Kim J. I. Wheeler M. T. Heintzelman A. K. Weake V. M. Chapple C. (2019). Mutation of mediator subunit CDK 8 counteracts the stunted growth and salicylic acid hyperaccumulation phenotypes of an Arabidopsis MED 5 mutant. New Phytol. 223, 233–245. doi: 10.1111/nph.15741, PMID: 30756399

[ref60] Martel A. Ruiz-Bedoya T. Breit-Mcnally C. Laflamme B. Desveaux D. Guttman D. S. (2021). The ETS-ETI cycle: evolutionary processes and metapopulation dynamics driving the diversification of pathogen effectors and host immune factors. Curr. Opin. Plant Biol. 62:102011. doi: 10.1016/j.pbi.2021.102011, PMID: 33677388

[ref61] Meena M. Swapnil P. J. (2019). Regulation of WRKY genes in plant defence with beneficial fungus Trichoderma: current perspectives and future prospects. Arch. Phytopathol. Plant Protect. 52, 1–17. doi: 10.1080/03235408.2019.1606490

[ref62] Mishina T. E. Zeier J. J. (2006). The Arabidopsis flavin-dependent monooxygenase *FMO1* is an essential component of biologically induced systemic acquired resistance. Plant Physiol. 141, 1666–1675. doi: 10.1104/pp.106.081257, PMID: 16778014 PMC1533925

[ref63] Nawrath C. Metraux J. P. (1999). Salicylic acid induction-deficient mutants of Arabidopsis express PR-2 and PR-5 and accumulate high levels of camalexin after pathogen inoculation. Plant Cell 11, 1393–1404. doi: 10.2307/3870970, PMID: 10449575 PMC144293

[ref64] Pajerowska-Mukhtar K. M. Emerine D. K. Mukhtar M. S. (2013). Tell me more: roles of NPRs in plant immunity. Trends Plant Sci. 18, 402–411. doi: 10.1016/j.tplants.2013.04.004, PMID: 23683896

[ref65] Pandey R. Muèller A. Napoli C. A. Selinger D. A. Pikaard C. S. Richards E. J.. (2002). Analysis of histone acetyltransferase and histone deacetylase families of *Arabidopsis thaliana* suggests functional diversification of chromatin modification among multicellular eukaryotes. Nucleic Acids Res. 30, 5036–5055. doi: 10.1093/nar/gkf660, PMID: 12466527 PMC137973

[ref66] Peart J. R. Cook G. Feys B. J. Parker J. E. Baulcombe D. C. (2002). An EDS1 orthologue is required for N-mediated resistance against tobacco mosaic virus. Plant J. 29, 569–579. doi: 10.1046/j.1365-313X.2002.029005569.x 11874570

[ref67] Peng Y. Van Wersch R. Zhang Y. (2018). Convergent and divergent signaling in PAMP-triggered immunity and effector-triggered immunity. Mol. Plant Microbe Interact. 31, 403–409. doi: 10.1094/MPMI-06-17-0145-CR, PMID: 29135338

[ref68] Peng Y. Yang J. Li X. Zhang Y. J. (2021). Salicylic acid: biosynthesis and signaling. Annu. Rev. Plant Biol. 72, 761–791. doi: 10.1146/annurev-arplant-081320-092855 33756096

[ref69] Qi G. Chen H. Wang D. Zheng H. Tang X. Guo Z.. (2021). The BZR1-EDS1 module regulates plant growth-defense coordination. Mol. Plant 14, 2072–2087. doi: 10.1016/j.molp.2021.08.011, PMID: 34416351

[ref70] Rate D. N. Greenberg J. T. (2001). The Arabidopsis aberrant growth and death2 mutant shows resistance to *Pseudomonas syringae* and reveals a role for NPR1 in suppressing hypersensitive cell death. Plant J. 27, 203–211. doi: 10.1046/j.0960-7412.2001.1075umedoc.x, PMID: 11532166

[ref71] Rietz S. Stamm A. Malonek S. Wagner S. Becker D. Medina-Escobar N.. (2011). Different roles of Enhanced Disease Susceptibility1 (EDS1) bound to and dissociated from Phytoalexin Deficient4 (PAD4) in Arabidopsis immunity. New Phytol. 191, 107–119. doi: 10.1111/j.1469-8137.2011.03675.x, PMID: 21434927

[ref72] Rochon A. Boyle P. Wignes T. Fobert P. R. Després C. (2006). The coactivator function of Arabidopsis NPR1 requires the core of its BTB/POZ domain and the oxidation of C-terminal cysteines. Plant Cell 18, 3670–3685. doi: 10.1105/tpc.106.046953, PMID: 17172357 PMC1785396

[ref73] Saile S. C. Jacob P. Castel B. Jubic L. M. Salas-Gonzalez I. Bäcker M.. (2020). Two unequally redundant “helper” immune receptor families mediate *Arabidopsis thaliana* intracellular “sensor” immune receptor functions. PLoS Biol. 18:e3000783. doi: 10.1371/journal.pbio.3000783, PMID: 32925907 PMC7514072

[ref74] Saleem M. Fariduddin Q. Castroverde C. Danve M. (2021). Salicylic acid: A key regulator of redox signalling and plant immunity. Plant Physiol. Biochem. 168, 381–397. doi: 10.1016/j.plaphy.2021.10.011, PMID: 34715564

[ref75] Saleh A. Withers J. Mohan R. Marqués J. Gu Y. Yan S.. (2015). Post-translational modifications of the master transcriptional regulator NPR1 enable dynamic but tight control of plant immune responses. Cell Host Microbe 18, 169–182. doi: 10.1016/j.chom.2015.07.005, PMID: 26269953 PMC4537515

[ref76] Savary S. Willocquet L. Pethybridge S. J. Esker P. Mcroberts N. Nelson A. J. (2019). The global burden of pathogens and pests on major food crops. Nat. Ecol. E *vol*. 3, 430–439. doi: 10.1038/s41559-018-0793-y, PMID: 30718852

[ref77] Sinha S. K. Kumar K. R. R. (2021). “Function of mediator in regulating salicylic acid mediated signaling and responses in plants,” in Jasmonates and Salicylates Signaling in Plants. eds. Aftab Tariq Yusuf Mohammad (Cham: Springer), 265–279.

[ref78] Spoel S. H. Loake G. J. (2011). Redox-based protein modifications: the missing link in plant immune signalling. Curr. Opin. Plant Biol. 14, 358–364. doi: 10.1016/j.pbi.2011.03.007, PMID: 21454121

[ref79] Spoel S. H. Mou Z. Tada Y. Spivey N. W. Genschik P. Dong X. (2009). Proteasome-mediated turnover of the transcription coactivator NPR1 plays dual roles in regulating plant immunity. Cell 137, 860–872. doi: 10.1016/j.cell.2009.03.038, PMID: 19490895 PMC2704463

[ref80] Sun X. Lapin D. Feehan J. M. Stolze S. C. Kramer K. Dongus J. A.. (2021). Pathogen effector recognition-dependent association of NRG1 with EDS1 and SAG101 in TNL receptor immunity. Nat. Commun. 12, 3335–3315. doi: 10.1038/s41467-021-23614-x, PMID: 34099661 PMC8185089

[ref81] Torrens-Spence M. P. Bobokalonova A. Carballo V. Glinkerman C. M. Pluskal T. Shen A.. (2019). PBS3 and EPS1 complete salicylic acid biosynthesis from isochorismate in Arabidopsis. Mol. Plant 12, 1577–1586. doi: 10.1016/j.molp.2019.11.005, PMID: 31760159

[ref82] Verger A. Monté D. Villeret V. (2019). Twenty years of mediator complex structural studies. Biochem. Soc. Trans. 47, 399–410. doi: 10.1042/BST20180608, PMID: 30733343 PMC6393861

[ref83] Vlot A. C. Dempsey D. M. A. Klessig D. F. (2009). Salicylic acid, a multifaceted hormone to combat disease. Annu. Rev. Phytopathol. 47, 177–206. doi: 10.1146/annurev.phyto.050908.135202, PMID: 19400653

[ref84] Wagner S. Stuttmann J. Rietz S. Guerois R. Brunstein E. Bautor J.. (2013). Structural basis for signaling by exclusive EDS1 heteromeric complexes with SAG101 or PAD4 in plant innate immunity. Cell Host Microbe 14, 619–630. doi: 10.1016/j.chom.2013.11.006 24331460

[ref85] Wang W. Chen X. (2004). HUA ENHANCER3 reveals a role for a cyclin-dependent protein kinase in the specification of floral organ identity in Arabidopsis. Development 131, 3147–3156. doi: 10.1242/dev.01187, PMID: 15175247 PMC5142244

[ref86] Wang Y. Schuck S. Wu J. Yang P. Döring A. C. Zeier J.. (2018). A MPK3/6-WRKY33-ALD1-pipecolic acid regulatory loop contributes to systemic acquired resistance. Plant Cell 30, 2480–2494. doi: 10.1105/tpc.18.00547, PMID: 30228125 PMC6241261

[ref87] Wang C. Yao J. Du X. Zhang Y. Sun Y. Rollins J. A.. (2015). The Arabidopsis mediator complex subunit16 is a key component of basal resistance against the necrotrophic fungal pathogen *Sclerotinia sclerotiorum*. Plant Physiol. 169, 856–872. doi: 10.1104/pp.15.00351, PMID: 26143252 PMC4577384

[ref88] Warren G. Mckown R. Marin A. Teutonico R. (1996). Isolation of mutations affecting the development of freezing tolerance in *Arabidopsis thaliana* (L.) Heynh. Plant Physiol. 111, 1011–1019. doi: 10.1104/pp.111.4.1011, PMID: 8756493 PMC160972

[ref89] Waszczak C. Akter S. Jacques S. Huang J. Messens J. Van Breusegem F. (2015). Oxidative post-translational modifications of cysteine residues in plant signal transduction. J. Exp. Bot. 66, 2923–2934. doi: 10.1093/jxb/erv084, PMID: 25750423

[ref90] Wathugala D. L. Hemsley P. A. Moffat C. S. Cremelie P. Knight M. R. Knight H. (2012). The mediator subunit SFR6/MED16 controls defence gene expression mediated by salicylic acid and jasmonate responsive pathways. New Phytol. 195, 217–230. doi: 10.1111/j.1469-8137.2012.04138.x, PMID: 22494141

[ref91] Wiermer M. Feys B. J. Parker J. E. (2005). Plant immunity: the EDS1 regulatory node. Curr. Opin. Plant Biol. 8, 383–389. doi: 10.1016/j.pbi.2005.05.010, PMID: 15939664

[ref92] Wildermuth M. C. Dewdney J. Wu G. Ausubel F. M. (2001). Isochorismate synthase is required to synthesize salicylic acid for plant defence. Nature 414, 562–565. doi: 10.1038/35107108, PMID: 11734859

[ref93] Withers J. Dong X. (2016). Post-translational modifications of NPR1: a single protein playing multiple roles in plant immunity and physiology. PLoS Pathog. 12:e1005707. doi: 10.1371/journal.ppat.1005707, PMID: 27513560 PMC4981451

[ref94] Yadav S. K. Srivastava D. (2017). “Biotic stress management in rice through RNA interference,” in Biotic Stress Management in Rice. eds. Shamim Md. Singh K. N. (Florida, USA: Apple Academic Press), 363–394.

[ref95] Yang L. Chen X. Wang Z. Sun Q. Hong A. Zhang A.. (2020). HOS15 and HDA9 negatively regulate immunity through histone deacetylation of intracellular immune receptor NLR genes in Arabidopsis. New Phytol. 226, 507–522. doi: 10.1111/nph.16380, PMID: 31854111 PMC7080574

[ref96] Yang L. Teixeira P. J. P. L. Biswas S. Finkel O. M. He Y. Salas-Gonzalez I.. (2017). *Pseudomonas syringae* type III effector HopBB1 promotes host transcriptional repressor degradation to regulate phytohormone responses and virulence. Cell Host Microbe 21, 156–168. doi: 10.1016/j.chom.2017.01.003, PMID: 28132837 PMC5314207

[ref97] Yang D. L. Yao J. Mei C. S. Tong X. H. Zeng L. J. Li Q.. (2012). Plant hormone jasmonate prioritizes defense over growth by interfering with gibberellin signaling cascade. PNAS 109, E1192–E1200. doi: 10.1073/pnas.1201616109, PMID: 22529386 PMC3358897

[ref98] Yu X. Xu Y. Yan S. (2021). Salicylic acid and ethylene coordinately promote leaf senescence. J. Integr. Plant Biol. 63, 823–827. doi: 10.1111/jipb.13074, PMID: 33501782

[ref99] Yuan M. Ngou B. P. M. Ding P. Xin X. F. (2021). PTI-ETI crosstalk: an integrative view of plant immunity. Curr. Opin. Plant Biol. 62:102030. doi: 10.1016/j.pbi.2021.102030, PMID: 33684883

[ref100] Zavaliev R. Mohan R. Chen T. Dong X. (2020). Formation of NPR1 condensates promotes cell survival during the plant immune response. Cell 182, 1093–1108.e18. doi: 10.1016/j.cell.2020.07.016, PMID: 32810437 PMC7484032

[ref102] Zhang X. Yao J. Zhang Y. Sun Y. Mou Z. (2013). The A rabidopsis M ediator complex subunits MED 14/SWP and MED 16/SFR 6/IEN 1 differentially regulate defense gene expression in plant immune responses. Plant J. 75, 484–497. doi: 10.1111/tpj.12216, PMID: 23607369

[ref103] Zhu Y. Schluttenhoffer C. M. Wang P. Fu F. Thimmapuram J. Zhu J. K.. (2014). CYCLIN-DEPENDENT KINASE8 differentially regulates plant immunity to fungal pathogens through kinase-dependent and-independent functions in Arabidopsis. Plant Cell 26, 4149–4170. doi: 10.1105/tpc.114.128611, PMID: 25281690 PMC4247566

